# A Luciferase-Based Approach for Functional Screening of 5′ and 3′ Untranslated Regions of the mRNA Component for mRNA Vaccines

**DOI:** 10.3390/vaccines13050530

**Published:** 2025-05-16

**Authors:** Maria Rubtsova, Yuliana Mokrushina, Dmitry Andreev, Maria Poteshnova, Nikita Shepelev, Mariya Koryagina, Ekaterina Moiseeva, Diana Malabuiok, Yury Prokopenko, Stanislav Terekhov, Aleksander Chernov, Elena Vodovozova, Ivan Smirnov, Olga Dontsova, Alexander Gabibov, Yury Rubtsov

**Affiliations:** 1 Shemyakin and Ovchinnikov Institute of Bioorganic Chemistry, Russian Academy of Science, 117997 Moscow, Russia; mprubtsova@gmail.com (M.R.); yuliana256@mail.ru (Y.M.); cycloheximide80@gmail.com (D.A.); evmoise@gmail.com (E.M.); malabuiokdiana@gmail.com (D.M.); tetrahydrofuran@mail.ru (Y.P.); sterekhoff@mail.ru (S.T.); alexandrchernov1984@gmail.com (A.C.); elvod.ibch@yandex.ru (E.V.); ivansmr@inbox.ru (I.S.); olga.a.dontsova@gmail.com (O.D.); 2 Department of Chemistry, Lomonosov Moscow State University, 119991 Moscow, Russia; mariia.poteshnova@chemistry.msu.ru (M.P.); nikita.shepelev96@gmail.com (N.S.); tais29998@mail.ru (M.K.); 3 Endocrinology Research Center of the Ministry of Health of the Russian Federation, 117292 Moscow, Russia; 4 Center of Life Sciences, Skolkovo Institute of Science and Technology, 143025 Moscow, Russia

**Keywords:** mRNA vaccine, translation, UTRs, secreted nanoluciferase, lipid nanoparticles, immunization, SARS-CoV-2, S protein

## Abstract

**Background/Objectives**: The recent COVID-19 pandemic caused by SARS-CoV-2 infection has highlighted the need for protocols for rapid development of efficient screening methods to search for the optimal mRNA vaccine structures against mutable viral agents. The unmatched success of mRNA vaccines by Pfizer and Moderna encoding the spike protein of SARS-CoV-2 confirms the potential of lipid nanoparticles for mRNA delivery for an accelerated development of new vaccines. The efficacy of vaccination and the production cost of mRNA-based vaccines largely depend on the composition of mRNA components, since the synthesis of an immunogenic protein requires precise and efficient translation *in vivo*. The composition of 5′ and 3′ UTR combinations of mRNA has a strong impact on the translation efficiency. The major objective of this study was to increase the probability of producing the immunogenic protein encoded by vaccine mRNA. For this purpose, we proposed to find a new combination of natural UTRs and, in parallel with that, to design and test the system for *in vivo* selection of translationally active UTRs. **Methods**: By using Ribo-Seq analysis, sets of candidate short UTRs were generated. These UTRs were tested both in cell cultures and in mice for effective production of secreted nanoluciferase (NLuc) and the S protein of SARS-CoV-2. A combination of the most effective UTRs was used to generate a prototype of an mRNA vaccine capable of inducing neutralizing antibodies against coronavirus. **Results**: The usefulness of the selected UTRs for vaccine development was tested by implicating the full-length coding sequence of SARS-CoV-2 S protein to produce the main immunogen. As a result, the system for functional screening of UTRs was created by using the NLuc gene. **Conclusions**: The proposed approach allows non-invasive quantitative assessment of the translational activity of UTRs in the blood serum of mice. By using the full-length sequence of SARS-CoV-2 S protein as a prototype, we demonstrated that the combination of UTRs selected using our luciferase-based reporter assay induces IgG titers and neutralization rates comparable to those obtained by using UTRs from commercial S-protein-based mRNA vaccines.

## 1. Introduction

Rapid urbanization and growing density of population, as well as human invasion of various environmental niches and other factors, have facilitated the transfer of viruses and other pathogens from animals to humans [1]. These factors significantly increase the development of new animal viruses that, after mutagenesis, can infect humans [2]. The most glaring example of these unwanted events is the global COVID-19 pandemic caused by SARS-CoV-2 [3]. A lack of information regarding the structure of the new virus, the speed of aerial spreading, and the infection rate led to rapid dissemination of the virus, overburdened healthcare systems, and long lockdown periods. The isolation of people had a negative economic impact and ultimately affected millions of people. Luckily, COVID-19 killed less than 2% of infected individuals [4]. The impact of COVID-19 on civilization set a goal for researchers and Big Pharma to rapidly produce a vaccine for mass immunization and drugs against SARS-CoV-2. The “vaccine race” was won by vaccines based on lipid nanoparticles “loaded” with the S protein mRNA [5]. This new type of vaccine and the revolutionary mode of delivery are based on “packing” the S protein mRNA into dense particles consisting of the mRNA itself and a combination of lipids [6]. These lipid nanoparticles, or LNPs, enter the cell cytoplasm upon intramuscular administration and release the mRNA, based on which the S protein is produced. The capped mRNA used for “packaging” is obtained by in vitro transcription and is modified with pseudouridine residues [7]. Due to the production of S protein following vaccination, immunogenic peptides derived from S protein are presented to T cells. This induces the production of anti-spike immunoglobulins and differentiation of memory CD4/CD8 T cells, as well as B and plasma cells [8]. The local response is sufficient for developing immunity against SARS-CoV-2, which mitigates the pathogenicity of the coronavirus and protects from severe COVID-19. The effectiveness and safety of mRNA vaccines based on LNPs proved the potential usefulness and power of this vaccine type. Despite early success against the initial or Wuhan variant of the coronavirus, the rapidly evolving new mutated variants of the virus were able to evade neutralization by antibodies elicited by the Wuhan-type S protein [9].

The immunogenicity of mRNA vaccines largely depends on mRNA stability, its translational competence, and the purity of mRNA packaged into LNPs. The mRNA stability can be improved by adding a cap structure to its 5′ end, modifying or blocking its 3′ end, and mRNA cyclization [10]. High purity and pseudouridine modification led to a lower production of type I interferon blocking translation [11]. The translation efficiency of vaccine mRNA strongly depends on the structure of 5′ and 3′ UTRs (untranslated regions).

We have selected a set of natural UTRs and proposed a protocol for their simple testing in cell lines and in vivo in laboratory mice. The testing protocol is based on the mRNA of NLuc (Promega), a secreted luciferase variant. Our protocol is suitable for assessing the translational competence of UTRs by analyzing NLuc [12] activity in culture media following transfection of cells with an expression cassette containing NLuc and UTRs. Using this protocol, we compared the translational activity of UTRs and chose the best ones. A combination of UTRs selected in vitro and named NEW-UTR was used with SARS-CoV-2 S protein mRNA for vaccination of mice and generated high titers of specific anti-spike antibodies with the virus-neutralizing activity in vitro. Overall, here we have proposed and successfully tested a new protocol for assessing the translational activity of UTRs suitable for mRNA vaccines. This protocol is useful for optimizing the non-coding regions of mRNA vaccines.

## 2. Materials and Methods

### 2.1. Preparation of Reporter Constructs

The following approach was used to obtain reporter constructs. Each structure was assembled from three fragments using the Gibson method [12] (Gibson, Young et al. 2009). Fragment 1 (F1) corresponded to the tested 5′ leader, while fragment 2 (F2) corresponded to the sequence of the secreted NLuc (this universal fragment was used in all assemblies), and fragment 3 (F3) corresponded to the 3′ UTR sequence. Fragment 1 contained the following sequences:

5′aaactgcccacttggcagtacatcaagtgtatcatatgCGCCGTAATACGACTCACTATAGGGAGCTTATCGATACCGTCGAGATCT-NNNN **ATGAACTCCTTCTCCACAAGCGCCTTCGGTCCAGTTGCCTTCTCCCTAGGCCTGCTCCTG**

The T7 promoter is underlined, N is the sequence of the tested UTR, and the sequence of the overlap with F2 is in bold.

Fragment 3 contained the following sequences:

5′ **TCAACGGAGTGACCGGCTGGCGGCTGTGCGAACGCATTCTGGCGTGATGA**GGCGCC-NNNN-CAATTGCCATTATAAGCTGCAATAAACAAGTTGcggccgcgatatctcgacaatcaacctctggattaca

The T7 promoter is underlined, N is the sequence of the tested UTR, and the sequence of the overlap with F2 is in bold. The corresponding fragments were synthesized by IDT. For the assembly, 1 µL of each fragment (F1, F2, and F3) with a concentration of 50 nM was mixed with 3 µL of Gibson Assembly Master Mix (E2611L, NEB) and incubated at 50 °C for 15 min. After completion of the reaction, a PCR was performed using the primers VAC 5′ ACTTGGCAGTACATCAAGTGTATCATATGCGCCG 3′ (anneals to the T7 F1 promoter) and FLA50 5′ TTTTTTTTTTTTTTTTTTTTTTTTTTTTTTTTTTTTTTTTTTTTTTTTAACTTGTTTATTGCAGCTTATAATGG’ (introduction of polyA (50), anneals to 3′ UTR in F3). A PCR was carried out using Q5^®^ High-Fidelity DNA Polymerase (NEB #M0491). The PCR products were verified by sequencing and used to obtain mRNA. The sequences of all UTRs are given in Supplementary Tables S1 and S2.

### 2.2. mRNA Synthesis and Purification

RNA was produced using RiboMAX™ Large Scale RNA Production Systems—T7 (Promega, Madison, WI, USA, Cat. # P1300). The following concentrations of triphosphates were used: cap analog m7GmAmG (Biolabmix, AGME-0050)—6 mM; N1-methylpseudouridine-5′-triphosphate (N1-methylpseudouridine, Biolabmix, Novosibirsk, Russia TNP-0050)—4 mM; rGTP (Promega, from the RiboMAX kit)—1.5 mM; rCTP and rATP (Promega, from the RiboMAX kit)—4 mM each. The reaction was performed according to the manufacturer’s recommendations for 3 h at 37 °C (the final volume was 10 µL for transfection into cells and 400 µL for in vivo mRNA LNP experiments). After treatment with DNase (from the RiboMAX kit), the mRNA was isolated using Monarch^®^ Spin RNA Cleanup Kit columns (NEB, T2030S).

### 2.3. Transfection of mRNA and Measurement of Luciferase Activity

THP-1 cells were cultured in RPMI 1640 medium with glutamine, penicillin/streptomycin, and 10% FBS (PanEco, Moscow, Russia). For transfection, the cells were seeded into a 48-well plate with a density of 4 × 10^5^ cells/mL. Each well was transfected with 200 ng of mRNA and 0.4 μL of Lipofectamine™ MessengerMAX (Thermo Fisher Scientific, Waltham, MA, USA, LMRNA015). In the indicated time intervals, 10 μL of conditioned medium was taken from the cells; 1 µL of the medium was used to measure NLuc activity using the Nano-Glo^®^ Luciferase Assay System kit (Promega, N1110) by GloMax^®^ 20/20 Luminometer (Promega).

### 2.4. Formulating and Characterizing mRNA LNPs

The content of components in the lipid mixture was similar to that described in the Pfizer-BioNTech COVID-19 Vaccine EUA Letter of Authorization (in mole percentage): ionizable lipid (ALC0315)–distearoylphosphatidylcholine (DSPC)–cholesterol–PEG lipid = 46.3%:9.4%:42.7%:1.6%. ALC0315 and PEG lipid (1,2-dimyristoyl-sn-glycero-3-methoxypolyethyleneglycol 2000) by BroadPharm (San Diego, CA, USA) and DSPC and cholesterol by Lipoid GmbH (Heidelberg, Germany) were used. mRNA LNPs were obtained by mixing an ethanol solution of lipids and an aqueous solution of mRNA in 100 mM sodium acetate (pH 5) in a microfluidic channel of the NanoGenerator Flex-M device (Suzhou Precigenome, Suzhou, China). The aqueous and ethanol solutions were mixed with a ratio of 3:1 by volume at a mixing speed of 5 mL/min. The mRNA was added at a concentration of 1 mg/mL and with the ratio of 1 mg of mRNA per 6 mL of LNPs. The resulting mRNA LNP suspension was immediately transferred to phosphate-buffered saline (PBS) with a ratio of 1:15 by volume in pre-washed Amicon^®^ Ultra-15 Centrifugal Filter Units, 30,000 MWCO. The average size of mRNA LNPs was analyzed using dynamic light scattering (Zetasizer Nano ZS, Malvern, UK).

RNA “loading” into LNPs was analyzed by measuring the level of the fluorescent signal when mRNA LNP suspensions were stained with RiboGreen reagent before and after their destruction in 2% Triton X-100 in PBS. The RNA concentration before and after mRNA LNP destruction was determined based on the calibration curves constructed during fluorescence measurements of RNA solutions of known concentration. Fluorescence measurements were carried out on a Victor X5 microplate reader (Perkin Elmer, Waltham, MA, USA) at an excitation wavelength of 485 nm and an emission wavelength of 535 nm.

### 2.5. RBD-Fc Expression and Purification

The codon-optimized DNA fragment encoding the S protein RBD of the SARS-CoV-2 strain Wuhan-Hu-1 (amino acid residues 331–528) was fused in frame by the overlap extension PCR method with the human IgG1 Fc region using specific primers. The following oligonucleotides were used: (1) RBD_FwEcoRI, 5′-aagagagaattccaatatcaccaatctgtgcc-3′; (2) RBD_Rv, 5′-cacaagatttgggctcgctagccttagggccgcacactg-3′; (3) Fc_Fw, 5′-gctagcgagcccaaatcttgtgacaaaactcacacatgcc-3′; and (4) Fc_RvAvrII, 5′-ttgagtcctaggtcatttacccggagacaggg-3′ (Fw and Rv indicate forward and reverse primers; introduced restriction sites EcoRI, NheI, and AvrII are underlined). The PCR product was digested with EcoRI/AvrII and cloned in frame with the interleukin-2 signal sequence at the EcoRI/NheI sites of the pFUSE-hIgG1-Fc2 vector (InvivoGen, San Diego, CA, USA). The resulting plasmid encoding RBD-Fc was used to transiently transfect FreeStyle 293-F cells in the serum-free FreeStyle 293 expression medium (Thermo Fisher Scientific, USA) using linear PEI 25K reagent (Polysciences, Warrington, PA, USA). Briefly, 1 µg of DNA per 1 mL of cells and PEI (1 mg/mL, pH 7) were separately diluted in FreeStyle medium with the ratio of 1:2, respectively, followed by addition of the DNA solution to the PEI mix and incubation for 30 min at room temperature. The cells were transfected at a density of 1 × 106 cells/mL and incubated at 37 °C with 8% CO_2_ and 135 rpm shaking for seven days. The culture medium was harvested by centrifugation (300× *g* for 10 min followed by 8,000× *g* for 15 min), adjusted into 1x phosphate-buffered saline (PBS, pH 7.2), and filtered through a 0.45 µm PES membrane. The recombinant RBD-Fc protein was purified by affinity chromatography in a HiTrap Protein G HP column (Cytiva, Marlborough, MA, USA) in accordance with the manufacturer’s protocol. The eluted protein was further purified by size exclusion chromatography on a Superdex 200 Increase column (GE Healthcare, Chicago, IL, USA) equilibrated in PBS. The protein concentration was measured spectrophotometrically using NanoDrop 2000 (Thermo Fisher Scientific, USA), and the purity was determined using 10% SDS-PAGE gel.

The recombinant RBD with C-terminal 6xHisTag was produced in HEK293-F cells as previously described [13]. Briefly, the recombinant RBD-6xHis protein was produced by transient expression in HEK293-F cells, followed by purification on a HiTrap Chelating HP column (Cytiva, USA) and gel filtration on a Superdex 75 10/300 GL (GE HealthCare, USA).

### 2.6. Experiments with Mice

Animals were housed under specific-pathogen-free conditions in the accredited IBCh animal breeding facility, with the quality management system certified for compliance with the international ISO:9001 standard (the Unique Research Unit Bio-Model of the IBCh, RAS; the Bioresource Collection–Collection of SPF-Laboratory Rodents for Fundamental, Biomedical and Pharmacological Studies, No. 075-15-2021-1067). BALB/cJ mice aged 6–9 weeks were used in experiments (https://www.jax.org/strain/000651#) (accessed on 1 January 2025). Animals were kept under controlled illumination (12:12 light–dark cycle) with water and standard combined fodder ad libitum. This study was approved by the Ethical Committee of the Shemyakin and Ovchinnikov Institute of Bioorganic Chemistry, Russian Academy of Sciences (protocol No. 369/2022). All procedures with animals were carried out in accordance with the approved study protocol.

The mice were divided into two groups for luciferase activity tests. NLuc mRNA was encapsulated in mRNA LNPs in PBS, or PBS was used to perform intramuscular injections for seven animals per group with an equivalent of 10 ug of mRNA. Four hours after the injection, blood was collected from the retro-orbital sinus and used for serum preparation. The serum was kept at −20 °C for future experiments.

For vaccination against SARS-CoV-2, three groups of mice were used: RBD-Fc in Alum, S protein (Wuhan) plus Pfizer UTRs, and S protein (Wuhan) plus NEW-UTRs. On day zero, the mice received intramuscular injections with the corresponding vaccine preparations (RBD-Fc—50 µg in 100 µL of PBS per mouse; S protein (Pfizer)—10 µg of RNA in 100 µL of PBS; S protein (NEW-UTR)—10 µg of RNA in 100 µL of PBS). On day 14, the mice were re-injected in accordance with the aforementioned protocol and kept for another two weeks. On day 30, blood was collected, and a specific anti-spike antibody titer was determined by ELISA. The neutralizing activity was measured using cellular tests with a pseudovirus-based platform.

### 2.7. Enzyme-Linked Immunosorbent Assay (ELISA)

To evaluate the antigen-specific IgG content in the serum from the immunized mice, the 96-well MaxiSorp plates (Nunc, Roskilde, Denmark) were coated with 100 ng of RBD-6xHiS protein per well in PBS at 4 °C overnight. The next day, the plates were washed five times with PBS containing 0.1% Tween 20 (PBST) and blocked with 0.1% sodium caseinate in PBST. The plates were incubated for one hour at room temperature with shaking at 450 rpm and then washed five times with PBST. The serum samples from individual mice were diluted 150 times in PBST with 0.225% sodium caseinate followed by diluting them three times in a serial manner. The plates were incubated for one hour at 37 °C with shaking and then washed five times with PBST. Goat Anti-Mouse IgG Antibody, Fc, HRP conjugate (Sigma Aldrich, Saint Louis, MO, USA, 63103, Cat. # AP127P) was added to each well in the form of a dilution of 1:70,000 in 0.225% sodium caseinate/PBST and incubated for one hour at 37 °C with shaking. After washing five times, tetramethylbenzidine (TMB) was added to the plates, which were then incubated for 15 min in the dark. The reaction was stopped by adding 1N phosphoric acid. The absorbance at 450 nm was measured by a Varioscan LUX microplate reader (Thermo Fisher Scientific, USA). Anti-RBD IgG titers were defined as the highest dilution factor whose OD450 exceeds a cut-off value. The cut-off value was determined as the mean of OD450 values of mock-immunized mouse sera plus three standard deviations. The experiment was performed in duplicate and averaged.

### 2.8. Pseudovirus Production and Neutralization Assay

Lentiviral pseudovirus production and neutralization were performed as previously described [14], with some modifications. Briefly, HEK293T cells and HEK293T-hACE2 cells stably expressing human ACE2 were cultured in DMEM (Thermo Fisher Scientific, USA), supplemented with 10% fetal bovine serum (HyClone Characterized FBS, Cytiva, USA), 2 mM GlutaMAX Supplement (Thermo Fisher Scientific, USA), and 1x Antibiotic-Antimycotic (Thermo Fisher Scientific, USA) in a humidified incubator at 37 °C with 8% CO_2_. The SARS-CoV-2 spike pseudotyped lentiviruses were generated by the co-transfection of HEK293T cells with a spike-encoding plasmid pCG1_SARS_2_S_dF_dc19 (1), a lentiviral packaging plasmid pMDLg_pRRE (2, Addgene ID: 12251), pRSV_Rev (3, Addgene ID: 12253), and a transfer plasmid encoding firefly luciferase pCDH-luc2 (4) at the ratio of 1:6:2:6, respectively, using linear PEI 25K in DNA–PEI ratio of 1:2. Following incubation at 37 °C and 8% CO_2_ for 72 h, the culture medium containing pseudovirus particles was harvested, clarified by centrifugation at 4000× *g* for 15 min at 4 °C, and filtered through a 0.45 µm PES membrane. Pseudovirus stocks in single-use aliquots were prepared and stored at −80 °C. One aliquot was titrated by twofold serial dilution to achieve a luminescence signal of ∼350,000 relative light units (RLUs).

For neutralization experiments, HEK293T-ACE2 cells were seeded at a density of 2 × 104 cells per well into 96-well flat-bottom cell culture plates (Nest, Wuxi, China) and incubated overnight at 37 °C with 8% CO_2_. The next day, when the cells reached ~80% confluence, the culture medium was carefully aspirated, and the serum samples were diluted three times in a row in DMEM + 10% FBS and transferred to cells, followed by infection with pseudoviruses. Serum sample dilution was selected based on antibody titers and covered a range of 1/120 to 1/787 320, each sample was prepared in a technical duplicate. Only pseudoviruses and serum samples from mock-immunized mice, encompassing the range of 1/120 to 1/29 160, were used as infection control. SARS-CoV-2-neutralizing monoclonal antibody P4A1 [15] was used as a positive control for inhibition of infection. Non-infected HEK293T-ACE2 wells in triplicate were used for assessing the luminescence background. The plates were incubated at 37 °C and 8% CO_2_ for 48 h, the culture medium was aspirated, and the cells were lysed using the Bright-Glo Luciferase Assay System (Promega, USA). Luminescence was quantified using a white 96-well microplate (Greiner Bio-One, **Kremsmünster,** Austria) on a Varioskan LUX microplate reader (Thermo Fisher Scientific, USA). All the experiments were repeated at least twice. The half-maximal neutralizing antibody titer (NT50) was calculated based on the neutralization curves by four-parameter non-linear regression in GraphPad Prism 10.4.0 software.

### 2.9. Statistical Analysis

GraphPad Prism 10.4.0 software was used to perform the complete statistical analysis. Antigen-specific IgG titers and NT50 values were presented as a geometric mean ± geometric standard deviation. Non-parametric Kruskal–Wallis one-way ANOVA followed by Dunn’s post hoc test was used for multiple comparisons. *p*-values less than 0.05 were considered statistically significant.

## 3. Results

### 3.1. Testing of 5′ Leaders and 3′ UTRs In Vitro

Initially, the translational activity of different 5′ and 3′ untranslated regions of mRNA was compared with the untranslated regions of the Pfizer BNT162b2 vaccine or Moderna mRNA-1273 vaccine against SARS-CoV-2. It was reasonable to use the Pfizer/Moderna UTRs and the S protein expression cassette as a gold standard. In test experiments, we decided to use much shorter mRNAs encoding secreted luciferase NLuc instead of S protein as the reporter gene. The advantage of these reporter mRNAs is that the samples of culture media conditioned by transfected cells could be directly analyzed at different time points. The number of reporter mRNAs at different time points allows us to reach a conclusion about the effectiveness of translation and the stability of particular mRNA in transfected cells.

**Figure 1:**
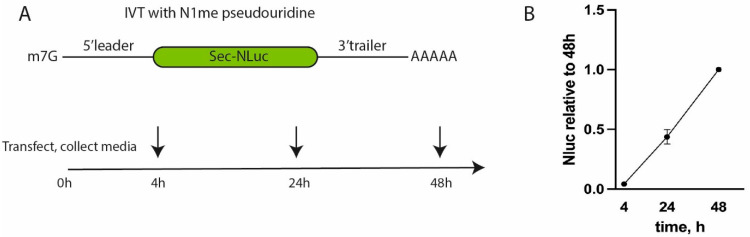
Testing of the reporter NLuc-expressing construct in THP-1 cells. (**A**) A scheme of the experiment with reporter mRNAs. (**B**) NLuc activity of mRNA with the 5′ leader and the 3′ UTR from Pfizer/BioNTech BNT162b2. Values are normalized to the activity at 48 h after transfection.

Firstly, we studied the influence of 5′ leaders from different cellular mRNAs on translation. Ribosome profiling or Ribo-Seq can map translating ribosomes on the cellular mRNAs with a precision of up to a single nucleotide [16,17]. In parallel, the mRNA was NGS-sequenced to profile the level of mRNAs. The ratio of Ribo-Seq/RNA-Seq signals shows how many ribosomes are “sitting” on particular mRNAs, which usually directly correlates with the translation efficiency of mRNA. 5′ UTRs from endogenous mRNAs were chosen according to the following criteria: (1) mRNA should be expressed at a high level; (2) mRNA should have a high Ribo-Seq/RNA-Seq index, which shows its translational activity; (3) the leader of mRNA should lack extra AUG codons; and (4) the mRNA leader should be unique. To estimate the translation efficiency, the data from [18] were used. Variants of the 5′ leader and the position of a transcription start site were determined using the data from [19]. Based on the above-mentioned criteria, the 5′ leaders of *FASN*, *CLTC*, *CTNNB1*, *GPI*, *HSPA1A*, *HSPA2*, and *MYH9* were selected for further study. The 5′ leader of *UCP2,* containing a short inhibitory reading frame, was employed [20] as a negative control. For comparison, the 5′ UTR from the mRNA-1273 vaccine [21] was added to the list (as a positive control). The corresponding reporter DNA constructs were generated, and reporter transcripts were synthesized by means of T7 transcription in vitro. The obtained RNAs were capped, polyadenylated (A50), and, importantly, all uridines were replaced with N1-methyl-pseudouridine. The mRNAs were transfected into the monocytic human cell line THP-1 [22], and the luciferase activity in the culture media was measured 4, 24, and 48 h post-transfection (Figure 1A).

Transfection with reference mRNA containing the 5′ leader and the 3′ UTR from Pfizer resulted in linear accumulation of NLuc according to the fluorescent signal in the interval from 4 to 48 h post-transfection (Figure 1B). At later time points, due to active cell growth, the culture medium was acidified; therefore, we decided to stop measurements to avoid potential inconsistency and artifacts. This result suggests high translation efficiency and stability of the corresponding reporter mRNA.

**Figure 2:**
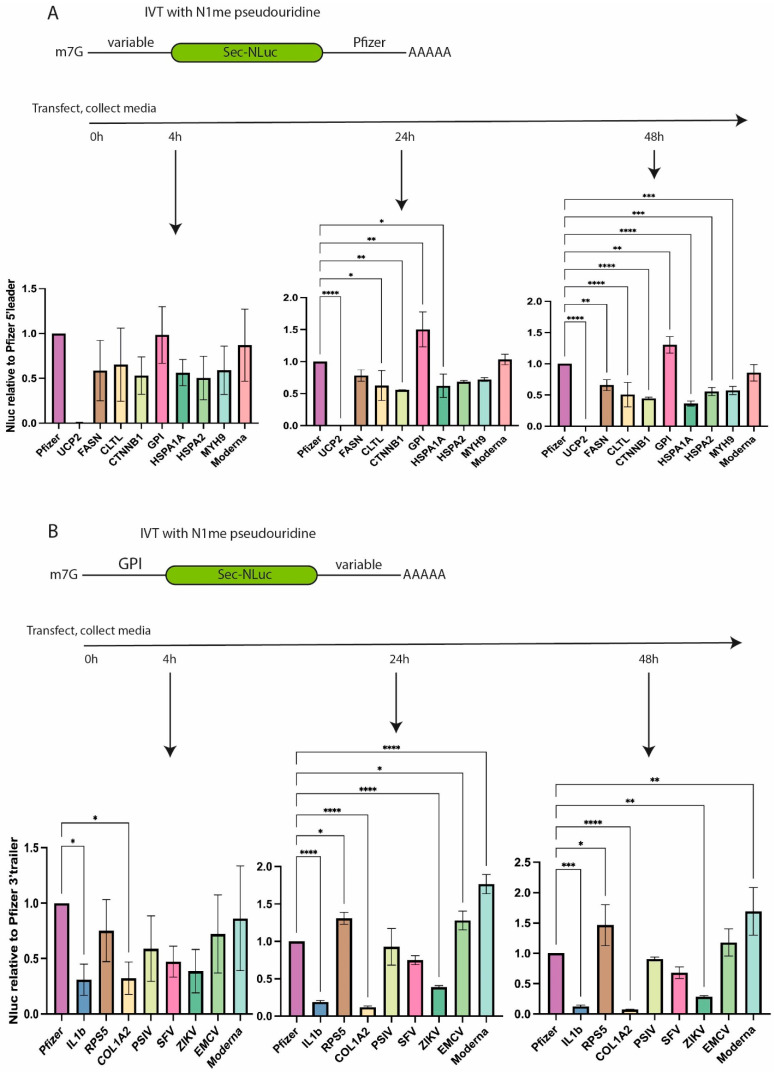
Translation efficiency of NLuc reporter mRNAs containing different UTRs according to the luciferase activity in the culture media of cells transfected with the corresponding reporter mRNAs. (**A**) Luciferase activity of mRNAs with different 5′ leaders at 4, 24, and 48 h post-transfection. Values are normalized to the signals obtained by using Pfizer 5′ leader and 3′ UTR in combination with NLuc. (**B**) Luciferase activity of mRNAs with different 3′ UTRs at 4, 24, and 48 h post-transfection. Values are normalized to the activity of NLuc mRNA containing the 5′ leader of *GPI* and the 3′ UTR of Pfizer. Normalized values are plotted as bar graphs with standard deviations of three independent experiments. * *p* < 0.05, ** *p* < 0.01, *** *p* < 0.001, **** *p* < 0.0001.

The mRNA containing tested 5′ UTRs showed a similar trend in luciferase activity to the control mRNA (Pfizer/BioNTech BNT162b2) at all three time points (Figure 2A). The efficacy of all 5′ UTRs (leader) except the negative control (UCP2) was comparable or slightly lower compared to 5′ UTR from Pfizer/BioNTech BNT162b2. This allows variants of native 5′ UTR to be chosen for further studies. The highest luciferase activity was found for the 5′ leader of glucose-6-phosphate isomerase (*GPI*). It demonstrated a slightly higher luciferase signal in two out of three experimental time points, namely 24 and 48 h, possibly due to the higher stability of mRNA and accumulation of NLuc in the media. Its 5′ leader is 64 nucleotides long and has an unusually low percentage of adenine nucleotides (6.25%).

The second set of experiments was performed to test different natural 3′ UTRs (Figure 2B). The literature was analyzed to pick several 3′ UTRs which could be responsible for mRNA stability in cells. Based on the mRNA stability data report [22], 3′ UTRs of *COL1A2* and *RPS5* were selected. Apart from cellular mRNAs, we decided to add several 3′ UTRs from viral RNAs. Viral 3′ UTRs stabilize viral mRNAs inside the host cells [23]. 3′ UTRs of viral RNAs from the encephalomyocarditis virus (EMCV), the Plautia stali intestine virus (PSIV), the Zika virus (ZIKV), and the Semliki Forest virus (SFV) were included in our analysis. Sequences of viral RNAs can contain unique cis-acting elements regulating mRNA stability such as ENE in the 3′ UTR of PSIV. This element binds to a poly(A) tail, forms a non-canonical triple helix, and inhibits mRNA deadenylation [24,25]. The 3′ UTR of *IL1b* which destabilizes mRNA in THP-1 cells [26] was chosen as a negative control.

mRNAs containing different variants of 3′ UTR and 5′ UTR from *GPI* were transfected to THP-1 cells, and luciferase activity was measured in the culture media at 4, 24, and 48 h post-transfection (Figure 2B). In contrast to the tested variants of the 5′ leaders, 3′ UTRs demonstrated a higher variability in luciferase activity, which was compared to the signal of reference mRNA containing the 3′ UTR from Pfizer/BioNTech BNT162b2. The least efficient mRNA contained 3′ UTR of IL1b and COL1A2. It is noteworthy that the ratio of signals from GPI-IL1B and GPI-COL1A2 mRNAs to the signal from the “GPI-Pfizer” reference mRNA decreased with time, possibly pointing to the destabilization of the corresponding mRNAs and a slowdown in luciferase production at later time points post-transfection (Supplementary Figure S1A). At the same time, the mRNA containing 3′ UTR from EMCV displays a tendency to increase luciferase expression compared to the control/reference mRNA, likely suggesting higher stability of this particular mRNA with EMCV 3′ UTR (Supplementary Figure S1A).

Four variants (Pfizer, Moderna, RPS5, and EMCV) of all tested 3′ UTRs demonstrated comparably high efficiency of NLuc production at different time points post-transfection (Figure 2B). To make a reasonable choice of the 3′ UTR, the HEK293T cell line was transfected with two out of four mRNAs, namely with the 3′ UTR from EMCV or from RPS5 (Supplementary Figure S1B). The translational activity of EMCV 3′ UTR-containing mRNA was higher than that of the one containing the 3′ UTR from RPS5 (Supplementary Figure S1B). Based on the obtained results, we selected the 5′ leader of GPI and the 3′ UTR of EMCV for further study.

### 3.2. Development of a Simplified In Vivo Testing System for Reporter mRNA

Transfection experiments with reporter mRNAs and cell cultures showed that NLuc activity in culture media is very high. Earlier, it was demonstrated that NLuc produced by tumor cells can be detected in the blood serum of mice [27]. We hypothesized that when the mice were treated with lipid nanoparticles containing mRNA encoding secreted NLuc, the activity of secreted luciferase in the serum would be sufficient for detection. mRNA LNPs containing reference mRNAs encoding NLuc with 5′ and 3′ UTRs of Pfizer were obtained using ionizing lipids for packing mRNA. The obtained particles had an average size of 68 nm, a polydispersity index (PDI) of 0.213, and carried 60.4 ng/μL of mRNA according to our measurements. Preliminary experiments failed to identify a correlation between LNP size and PDI, while amount of loaded mRNA was a factor. mRNA LNP preparations were injected into the thigh muscles of the mice, and blood was collected 4, 24, and 72 h post-infection to measure NLuc activity.

**Figure 3:**
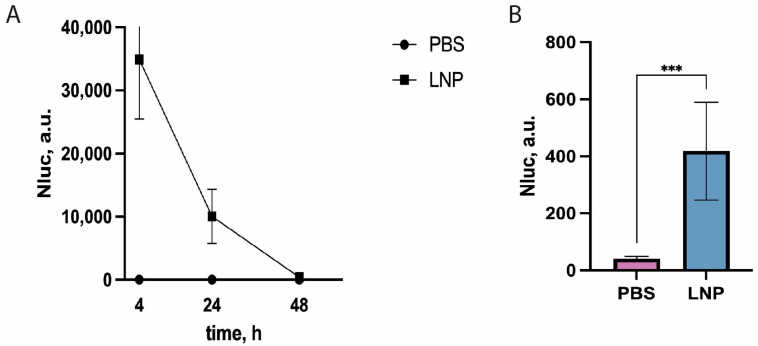
NLuc activity in mouse blood serum 4, 24, and 72 h post-intramuscular-administration of mRNA LNPs “loaded” with NLuc mRNA combined with UTRs from GPI and EMCV. (**A**) Time course of NLuc activity in mouse serum injected with mRNA LNPs. Mean values of triplicate measurements with standard deviations of one out of three biological replicates are shown (*n* = 5 in each independent experiment). Control—PBS. NLuc activity values 72 h post-injection are presented on the right plot. (**B**) Absolute values of NLuc activity 72 h post-injection. *** *p* < 0.001 according to Student’s test.

High luciferase activity was detected in the blood serum of the injected mice as soon as four hours post-administration of mRNA LNPs (Figure 3A). NLuc activity was three orders of magnitude higher than the activity in the negative control (PBS-injected mice). As expected, the luciferase activity decreased with time, but a reliable luminescent signal from NLuc activity was much higher than the background one even 72 h post-administration (Figure 3B). This result suggests that our protocol is suitable for fast and reliable measurement of relative efficacy of delivery and translational activity of reporter mRNA in vivo.

### 3.3. Testing of a New Combination of 5′ and 3′ UTR for Vaccine Development

**Figure 4:**
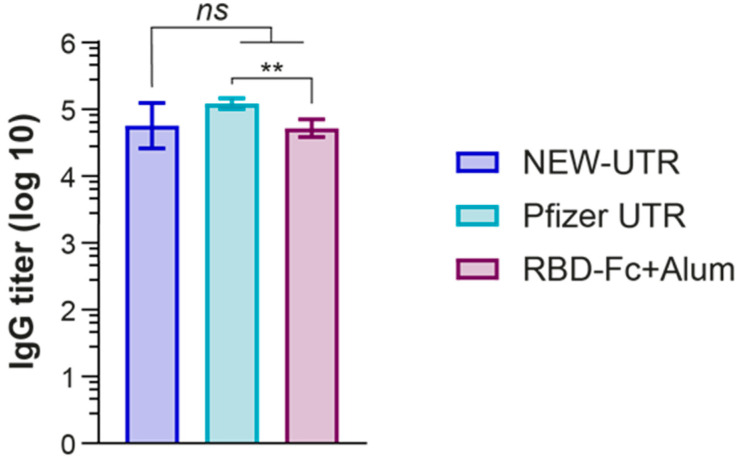
Humoral immune response induced by the NEW-UTR mRNA vaccine, Pfizer mRNA vaccine, and recombinant RBD-Fc protein with Alum as the adjuvant. Bars indicate the geometric mean titer (GMT) with the geometric standard deviation (GSD) = 7 mice per group. The statistical significance among the groups was analyzed by the Kruskal–Wallis test. ** *p* < 0.01; ns—not significant.

Luciferase tests of reporter mRNA containing a new combination of 5′ and 3′ UTRs of GPI-EMCV, which we named NEW-UTR, showed that it could potentially be used for generating mRNA-based vaccines. To prove this, we decided to insert the cDNA clone of the SARS-CoV-2 S protein between GPI 5′ and EMCV 3′ UTR and use the resulting mRNA to make mRNA LNPs and vaccinate mice against SARS-CoV-2. Plasmid DNA constructs—the experimental one containing NEW-UTR, and the control one containing Pfizer UTRs—were generated, and the corresponding mRNAs were transcribed in vitro and purified. The obtained mRNAs were efficiently packed into mRNA LNPs using the same set of lipids for Pfizer and NEW-UTR-containing mRNAs. Prior to injection, the mRNA content of mRNA LNPs (115.6 for NEW-UTR and 71.5 ng/μL for Pfizer UTR) was determined, and the average size (147.4 for NEW-UTR and 71.5 nm for Pfizer UTR) and size distribution (PDI 0.24 for both variants) of mRNA LNPs were measured. For vaccination, we used an amount of particle suspension equivalent to 10 μg of mRNA. As a positive control for vaccination, the RBD-Fc fusion protein and Alum as an adjuvant were used. Animals received two injections of mRNA vaccines and RBD-Fc as the control with an interval of 14 days. Blood was collected on day 14 after the second injection. Three groups of mice were used in the experiment: mice vaccinated with RBD-Fc as a positive control, mice vaccinated with mRNA encoding S protein with Pfizer UTRs in mRNA LNPs, and those vaccinated with mRNA encoding S protein with NEW-UTRs in mRNA LNPs. The humoral immune responses in the mice were analyzed using an endpoint ELISA. RBD was used as an antigen to detect the presence of specific antibodies in the serum samples as the main target for neutralizing antibodies. Analysis of blood serum revealed the presence of a high level of S-protein-specific antibodies according to ELISA (Figure 4). The antibody titer in the NEW-UTR group was higher than that in the RBD-Fc group, although it was slightly lower than in the Pfizer group.

**Figure 5:**
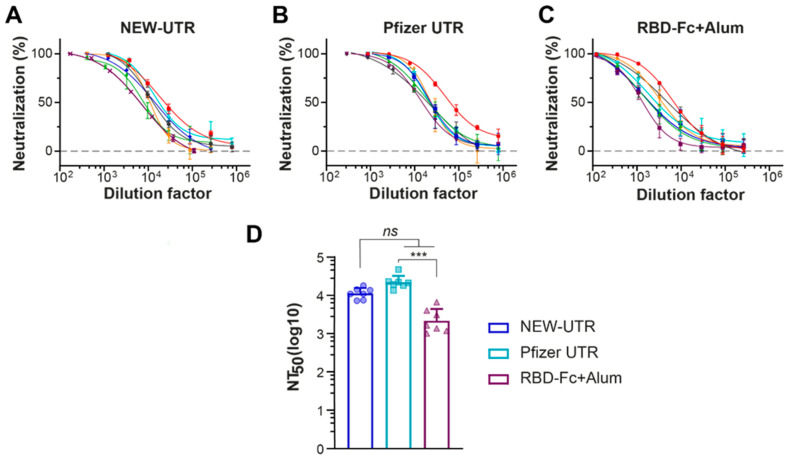
Neutralizing potency against SARS-CoV-2 pseudotyped lentiviruses of serum samples from mice immunized with mRNA LNPs. (**A**–**C**) Neutralization titration curves of serum samples from mice treated with NEW-UTR mRNA, Pfizer mRNA, and RBD-Fc with Alum as an adjuvant, respectively. Samples from individual mice are shown in different colors. (**D**) The half-maximal neutralizing antibody titer (NT_50_) values for individual mice calculated in GraphPad Prism software by four-parameter best-fit analysis are presented as dots and histograms. Bars indicate GMT with SD, *n* = 7 mice per group. The statistical significance among the groups was analyzed by the Kruskal–Wallis test. *** *p* < 0.001; ns—not significant.

Next, we examined whether the corresponding mRNAs with different UTRs, Pfizer versus NEW-UTR, would elicit production of substantial titers of neutralizing antibodies. As expected, in terms of the titer of S-protein-specific antibodies, Pfizer demonstrated the best result. While the titers of NEW-UTR were also lower than those of Pfizer, the level of neutralizing antibodies was still very high (Figure 5). Notably, this result was obtained on wild-type unoptimized sequences of UTRs. The results of S-protein-specific antibody production and the neutralizing activity further support the efficiency of our UTR screening protocol and prove its applicability for quick and reliable searching for translationally active combinations of UTRs for vaccine development.

## 4. Discussion

Selecting untranslated regions (UTRs) in mRNA vaccines has become necessary as a critical factor in optimizing vaccine efficacy, stability, tissue specificity, and immunogenicity. Recent advances in bioinformatics, synthetic biology, and artificial intelligence have enabled researchers to identify and engineer UTR sequences that enhance protein expression and immune responses. Concurrently, the rapid development of mRNA vaccines during the COVID-19 pandemic focused on UTR designs, lipid nanoparticle (mRNA LNP) delivery systems, and nucleotide modifications. UTRs regulate mRNA stability, subcellular localization, and translation efficiency by interacting with RNA-binding proteins and microRNAs.

The 5′ UTR contains ribosome binding sites and internal ribosome entry segments (IRES), while the 3′ UTR influences polyadenylation and degradation rates [28,29]. Optimal 5′ UTRs enhance protein yield by forming secondary structures that facilitate ribosomal scanning while avoiding premature initiation or stalling [28]. TOP (terminal oligopyrimidine) genes, such as GPI, represent a specialized class whose 5′ UTRs contain pyrimidine-rich tracts regulated by mTOR signaling, linking translational activity to cellular growth conditions [28].

Ideally, therapeutic mRNAs should be designed to produce the right amounts of encoded protein(s) in the desired cells, while being inactive in off-target cells. To maximize the therapeutic protein yield, early mRNA vaccines adopted UTRs from highly expressed human α-globin and β-globin to maximize antigen production. For example, the Pfizer/BioNTech BNT162b2 vaccine incorporated a 35-nucleotide fragment from the 5′ UTR of human α-globin, leveraging its evolutionary optimization for high translation efficiency [30]. The recent report from Bicknell et al. challenged this assumption [31]. It was demonstrated that attenuating ribosome load on mRNA improves S protein output by limiting translation-dependent mRNA decay. Therefore, paradoxically, 5′ UTRs that allow the most efficient translation initiation can be non-optimal for therapeutic mRNAs. Another confounding factor in the rational design of optimal UTRs is linked to the effect of nucleoside modifications, such as frequently used N1-methylpseudouridine, on mRNA translation and stability. Nucleoside-modified mRNAs can obtain various unpredicted properties as nucleotide modifications can alter various steps in mRNA decoding [32,33,34,35,36,37] and disrupt RNA cis-acting elements that are otherwise active in unmodified contexts [38,39]. This explains why certain cis-acting enhancer elements that are expected to substantially increase mRNA translation and stability or allow cell-specific translation are inactive in fully modified mRNAs.

Recent reports claim that cell-type-specific UTRs can further enhance the performance of vaccine mRNAs. A 2024 study identified thymosin β10 (TMSB10) as a gene with exceptionally high mRNA abundance in dendritic cells, making its UTRs prime candidates for vaccine optimization. Constructs combining 5′ and 3′ UTRs of TMSB10 boosted luciferase expression by a factor of 2.8 in antigen-presenting cells compared to traditional α-globin UTRs. In murine models, SARS-CoV-2 vaccines with TMSB10 UTRs elicited IgG titers against the Delta variant which were 4.3 times higher and enhanced CD4+/CD8+ T cell proliferation by 62% compared to controls [28]. These findings underscore the value of immune-cell-targeted UTR engineering.

The traditional approach to finding natural UTRs that enhance translation is based on juxtaposing transcriptomic and mass spectrometry data to identify highly abundant mRNAs, which presumably provide a high level of the corresponding proteins. However, this approach has several limitations. For instance, a protein encoded by highly expressed mRNA could be unstable due to rapid degradation. Translation of a particular mRNA could depend on nutritional or stress-related factors, further complicating the interpretation of the results. Another problem is that qualitative MS, which requires isotope-labeled reference peptides for precise measurements of protein levels, should be used. The above-mentioned issues could mostly be resolved by Ribo-Seq analysis, which relies on comparing transcriptomic data with profiles of mRNA, being associated with actively translating ribosomes. The ratio of the mRNA level to the level of actively translated messengers shows the translation efficiency per single mRNA. This means that Ribo-Seq technology is an excellent tool for mining data on actively translated mRNAs, likely containing functional UTRs.

Historically, 5′ UTRs are better studied than 3′ UTRs. Therefore, it is reasonable to combine both a translation-improving 5′ UTR from one gene/source with different 3′ UTRs in one reporter mRNA to search for the optimal combination. Generation of an effective mRNA vaccine requires testing the vaccine mRNA and its components in vitro and in vivo, with the latter being a time-limiting step. Therefore, the development of simple and rapid protocols for testing translationally active and stable mRNAs is still in high demand. Here, we utilized the advantage of the secreted NLuc variant of luciferase to set up consecutive screens of the mini library of Ribo-Seq-selected mRNAs for finding natural short 5′ UTRs, and then 3′ UTRs, which enhance translation of the reporter. The best combination of UTRs (5′ from human GPI gene and 3′ from EMCV viral RNA, named NEW-UTRs) was identified using these screens in vitro and then in vivo. For reference, we used the renowned combination of UTRs from the Pfizer SARS-CoV-2 S-protein-expressing vaccine and the corresponding set of packaging lipids to generate vaccine mRNA LNPs.

In vitro, the highest and comparable activity of the secreted NLuc was found in the culture media conditioned by the cells transfected with reporter NLuc mRNAs containing 5′ UTR from human GPI mRNA and α-globin leader from the Pfizer vaccine. We combined the 5′ UTR from GPI with the NLuc protein-coding sequence and different 3′ UTRs from a set of 3′ UTRs in one construct, which, according to our predictions, would stabilize or destabilize the reporter mRNA. In transfection experiments, we found that the best stability/translation efficiency can be achieved by using the 3′ UTR from an attenuated variant of EMCV. Therefore, we decided to combine UTRs of both GPI and EMCV RNAs in reporter NLuc-expressing constructs. To our great surprise, after intramuscular administration of small doses of NLuc-based reporters (5 micrograms in mRNA equivalent of NEW-UTR mRNA), the activity of NLuc was high in the serum of mice even several days after the injections. Even small aliquots of serum samples were sufficient for reliable detection of the luminescence in the period of several days. This observation demonstrates that NLuc-based mRNA reporters could be efficiently used to compare the translational activity of different mRNAs. The most striking conclusion is that even small amounts of NLuc-expressing mRNAs could be easily tested directly in mice. The translational competence of mRNAs in the tissue culture does not often reflect how it will be translated in animal models. Our finding provides an opportunity to test mRNA UTRs for mRNA vaccines and expression vectors for gene therapy directly in animals, avoiding tissue culture routine. In addition, analyzing secreted NLuc enzymatic activity is cheap, fast, and does not require additional processing of tissues compared with intracellular variants of firefly luciferase or similar reporters.

Finally, we tested the capacity of the NLuc-selected combination of the NEW-UTRs to drive translation of the long messenger, comparable to Pfizer/Moderna S-protein-expressing mRNA in terms of size in laboratory mice. Our results clearly show that the immune response to the vaccine assessed by specific IgG titers in the serum of the treated mice was comparable to the response caused by an analog of the Pfizer vaccine against the coronavirus. The neutralizing IgG titers in the serum samples from the immunized mice were also similar.

## 5. Conclusions

Taken together, we showed the feasibility of the new combination of UTRs for generating mRNA vaccines. Furthermore, it has been proven that the proposed NLuc-based protocol for simplified testing of translational competence of UTRs is an excellent option to search for new UTR variants of natural origin or generated computationally.

## Data Availability

Data are publicly available at DOI: https://doi.org/10.6084/m9.figshare.28638518; https://doi.org/10.6084/m9.figshare.28638476.v1; https://doi.org/10.6084/m9.figshare.28638467.v1; and https://doi.org/10.6084/m9.figshare.28638458.v1.

## Supplementary Materials

The following supporting information can be downloaded at: https://www.mdpi.com/article/10.3390/vaccines13050530/s1, Figure S1: Relative luciferase activity of mRNAs with different 3′and 5′ UTRs; Table S1: Sequences of 5′ leaders; Table S2: 3′UTR Sequences.
