# Supplementary material for: A Luciferase-Based Approach for Functional Screening of 5′ and 3′ Untranslated Regions of the mRNA Component for mRNA Vaccines
Source: Vaccines (Basel). 2025 May 16;13(5):530. doi: 10.3390/vaccines13050530 (PMC12115628; doi:10.3390/vaccines13050530)
Supplement: Supplementary file 1 [file vaccines-13-00530-s001.zip › vaccines-3573673-supplementary.pdf]

vaccines

## Supplementary Materials

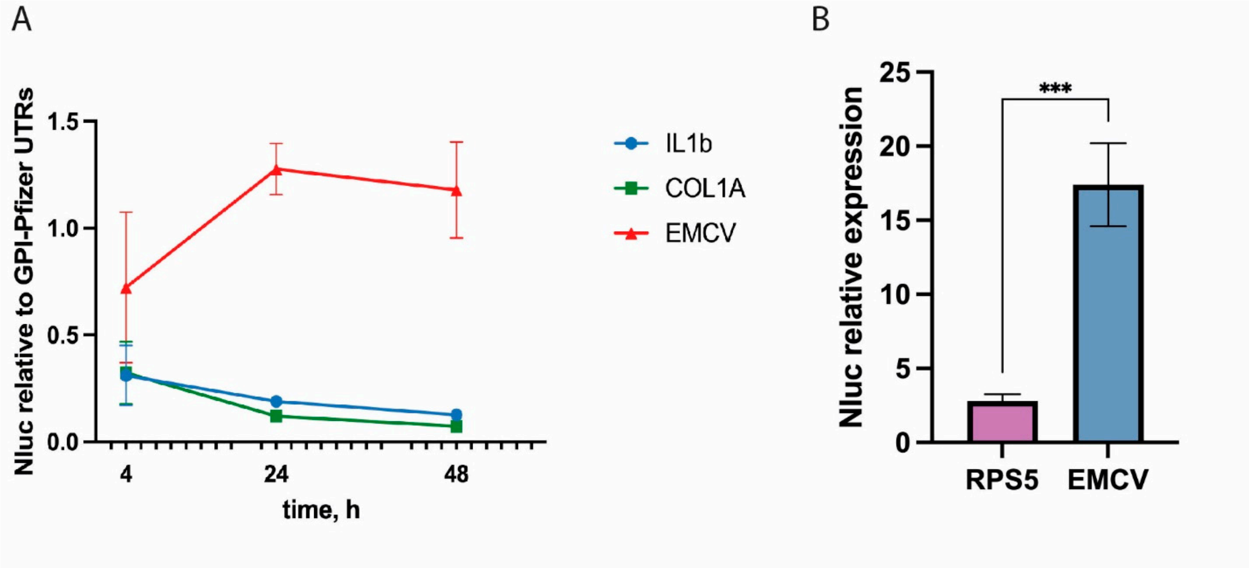


**Supplementary Figure S1.** A) Relative luciferase activity of mRNAs with 3'UTRs from IL1b, COL1A2 and EMCV after 4, 24 and 48 hours post transfection. Signal values were normalized to activity of mRNA with 5'leader of GPI and 3'UTR from Pfizer.; B) Relative luciferase activity of mRNA GPI-RPS5 and mRNA GPI-EMCV in HEK293T cells. Normalized values of luminescence signal at 4-, 24- and 48-hours post-transfection.

**Supplementary Table S1.** Sequences of 5'leaders

| Name | Reference | Nucleotide sequence |
|---|---|---|
| Pfizer | Vector BNT162b2 clone Vacc_Batch_FP8191, complete sequence PP544445.1 | AGAATAAACTAGTATTCTTCTGGTCCCCACAGACTCAGA-GAGAACCCGCCACC |
| UCP2 | Homo sapiens uncoupling protein 2 (UCP2), transcript | ACTGCGAAGCCCAGCTGCGCGCGCCTTGGGATTGACTGTC CACGCTCGCCCGGCTCGTCCGACGCGCCCTCCGCCAGCCG ACAGACACAGCCGCACGCACTGCCGTGTTCTCCCTGCGGC TCGGACACATAGTATGACCATTAGGTGTTTCGTCTCCCAC CCATTTTCTATGGAAAACCAAGGGGATCGGGCCATGATA |

| | | |
|---|---|---|
| | variant 8, mRNA NM_001381950.1 | GCCACTGGCAGCTTTGAAGAACGGGACACCTTTAGAGAAGCTTGATCTTGGAGGCCTCACCGTGAGACCTTACAAAGCCGGATTCCGGCAGAGTTCCTCTATCTCGTCTTGTTGCTGATTAAAGGTGCCCCTGTCTCCAGTTTTTCTCCATCTCCTGGGACGTAGCAGGAAATCAGCATC |
| FASN | Homo sapiens fatty acid synthase (FASN), mRNA NM_004104.5 | GAGCCAGAGAGACGGCAGCGGCCCCGGCCTCCCTCTCCGCCGCGCTTCAGCCTCCCGCTCCGCCGCGCTCCAGCCTCGCTCTCCGCCGCCCGCACCGCCGCCCGCGCCCTCACCAGAGCAGCC |
| CLTC | Homo sapiens clathrin heavy chain (CLTC), transcript variant 1, mRNA NM_004859.4 | TCCGCCATTGCGGCTCTCCTGGCCCCTGGAGCCTCCGCCCCCGACCCGAGCTCTTTCGTCTGCCTGCCAGTTTCCTGCGTCCCCGGAGAGGATCCTGCTGAGCCCAGCCTCCCCCCTCCCCTTCTCCTCCTCTCCCTTGGAGAGCCCGGGCAGCCACTGCCCCGCAGCCCCAGTGACAGGAGGAGACCATAACCCCCGACAGCGCC |
| CTNNB1 | Homo sapiens catenin beta 1 (CTNNB1), transcript variant 4, mRNA NM_001330729.2 | CTCTCGGTCTGTGGCAGCAGCGTTGGCCCGGCCCCGGGAGCGGAGAGCGAGGGGAGGCGGAGACGGAGGAAGGTCTGAGGAGCAGCTTCAGTCCCCGCCGAGCCGCCACCGCAGGTCGAGGACGGTCGGACTCCCGCGGCGGGAGGAGCCTGTTCCCCTGAGGGTATTTGAAGTATACCATACAACTGTTTTGAAAATCCAGCGTGGACA |
| GPI | Homo sapiens glucose-6-phosphate isomerase (GPI), transcript variant 7, mRNA NM_001329911.2 | GCGCGCTGCCGGCGCTCCTTCCTCCTCGGCTCGCGTCTCACTCAGTGTACCTTCTAGTCCCGCC |

| | | |
|---|---|---|
| HSPA1A | Homo sapiens heat shock protein family A (Hsp70) member 1A (HSPA1A), mRNA NM_005345.6 | AACGGCTAGCCTGAGGAGCTGCTGCGACAGTCCACTACCTTTTTCGAGAGTGACTCCCGTTGTCCCAAGGCTTCCCAGAGCGAACCTGTGCGGCTGCAGGCACCGGCGCGTCGAGTTTCCGGCGTCCGGAAGGACCGAGCTCTTCTCGCGGATCCAGTGTTCCGTTTCCAGCCCCCAATCTCAGAGCGGAGCCGACAGAGAGCAGGGAACCGGC |
| HSPA2 | Homo sapiens heat shock protein family A (Hsp70) member 2 (HSPA2), transcript variant 1, mRNA NM_021979.4 | GAGTTGCTGGTAGTGCCCGTGGTGCTTGGTTCGAGGTGGCCGTTAGTTGACTCCGCGGAGTTCATCTCCCTGGTTTTCCCGTCCTAACGTCGCTCGCCTTTCAGTCAGG |
| MYH9 | Homo sapiens myosin heavy chain 9 (MYH9), mRNA NM_002473.6 | GCGGTTCCTGGGCAGGGCACGGAAGGCTAAGCAAGGCTGACCTGCTGCAGCTCCCGCCTCGTGCGCTCGCCCCACCCGGCCGCCGCCCGAGCGCTCGAGAAAGTCCTCTCGGGAGAAGCAGCGCCTGTTCCCGGGGCAGATCCAGGTTCAGGTCCTGGCTATAAGTCACC |
| Moderna | Modern mRNA1273 expression vector, complete sequence OR134578.1 | GGGAAATAAGAGAGAAAAGAAGAGTAAGAAGAAATATAAGACCCCGGCGCCGCCACC |
| SECNLUC CDS | | ATGAACTCCTTCTCCACAAGCGCCTTCGGTCCAGTTGCCTTCTCCCTaGGCCTGCTCCTGGTGTTGCCTGCTGCCTTCCCTGCCCCAGTCTTCACACTCGAAGATTTCGTTGGGGACTGGCGACAGACAGCCGGCTACAACCTGGACCAAGTCCTTGAACAGGGAGGTGTGTCCAGTTTGTTTCAGAATCTCGGGGTGTCCGTAACTCCGATCCAAAGGATTGTCCTGAGCGGTGAAAAT |

| | | |
|---|---|---|
| | | GGGCTGAAGATCGACATCCATGTCATCATCCCGTATGAAG GTCTGAGCGGCGACCAAATGGGCCAGATCGAAAAAATTT TTAAGGTGGTGTACCCTGTGGATGATCATCACTTTAAGGT GATCCTGCACTATGGCACACTGGTAATCGACGGGGTTACG CCGAACATGATCGACTATTTCGGACGGCCGTATGAAGGC ATCGCCGTGTTCGACGGCAAAAAGATCACTGTAACAGGG ACCCTGTGGAACGGCAACAAAATTATCGACGAGCGCCTG ATCAACCCCGACGGCTCCCTGCTGTTCCGAGTAACCATCA ACGGAGTGACCGGCTGGCGGCTGTGCGAACGCATTCTGG CGTGATGA |

**Supplementary Table S2.** 3'UTR Sequences

| Name | Reference | Nucleotide sequence |
|---|---|---|
| Pfizer | Pfizer bivalent expression vector BNT162b2, complete sequence OR134577.1 | CTCGAGCTGGTACTGCATGCACGCAATGCTAGCTGCCCC TTTCCCGTCCTGGGTACCCCGAGTCTCCCCCGACCTCGG GTCCCAGGTATGCTCCCACCTCCACCTGCCCCACTCACC ACCTCTGCTAGTTCCAGACACCTCCCAAGCACGCAGCAA TGCAGCTCAAAACGCTTAGCCTAGCCACACCCCCACGGG AAACAGCAGTGATTAACCTTTAGCAATAAACGAAAGTTT AACTAAGCTATACTAACCCCAGGGTTGGTCAATTTCGTG CCAGCCACACCCTGGAGCTAGC |
| IL1b | Homo sapiens interleukin 1 beta (IL1B), mRNA NM_000576.3 | AGAGAGCTGTACCCAGAGAGTCCTGTGCTGAATGTGGAC TCAATCCCTAGGGCTGGCAGAAAGGGAACAGAAAGGTT TTTGAGTACGGCTATAGCCTGGACTTTCCTGTTGTCTACA CCAATGCCCAACTGCCTGCCTTAGGGTAGTGCTAAGAGG ATCTCCTGTCCATCAGCCAGGACAGTCAGCTCTCTCCTTT CAGGGCCAATCCCCAGCCCTTTTGTTGAGCCAGGCCTCT CTCACCTCTCCTACTCACTTAAAGCCCGCCTGACAGAAA CCACGGCCACATTTGGTTCTAAGAAACCCTCTGTCATTC GCTCCCACATTCTGATGAGCAACCGCTTCCCTATTTATTT ATTTATTTGTTTGTTTGTTTTATTCATTGGTCTAATTTATT CAAAGGGGGCAAGAAGTAGCAGTGTCTGTAAAAGAGCC TAGTTTTTAATAGCTATGGAATCAATTCAATTTGGACTG GTGTGCTCTCTTTAAATCAAGTCCTTTAATTAAGACTGAA AATATATAAGCTCAGATTATTTAAATGGGAATATTTATA AATGAGCAAATATCATACTGTTCAATGGTTCTGAAATAA ACTTCACTGAAGAAAAAAAAA |
| RPS5 | Homo sapiens ribosomal protein S5 | TTTTCCCAGCTGCTGCCCAATAAACCTGTCTGCCCTTTGG GGCAGTCCCAGCCA |

| | | |
|---|---|---|
| | (RPS5), mRNA NM_001009.4 | |
| COL1A2 | Homo sapiens collagen type I alpha 2 chain (COL1A2), mRNA NM_000089.4 | ATGAACTCAATCTAAATTAAAAAAGAAAGAAATTTGAAAAAACTTTCTCTTTGCCATTTCTTCTTCTTCTTTTTAACTGAAAGCTGAATCCTTCCATTTCTTCTGCACATCTACTTGCTTAAATTGTGGGCAAAAGAGAAAAAGAAGGATTGATCAGAGCATTGTGCAATACAGTTTCATTAACTCCTTCCCCCGCTCCCCCAAAAATTTGAATTTTTTTTTCAACACTCTTACACCTGTTATGGAAAATGTCAACCTTTGTAAGAAAACCAAAATAAAAATTGAAAAATAAAAACCATAAACATTTGCACCACTTGTGGCTTTTGAATATCTTCCACAGAGGGAAGTTTAAAACCCAAACTTCCAAAGGTTTAAACTACCTCAAAACACTTTCCCATGAGTGTGATCCACATTGTTAGGTGCTGACCTAGACAGAGATGAACTGAGGTCCTTGTTTTGTTTTGTTCATAATACAAAGGTGCTAATTAATAGTATTTCAGATACTTGAAGAATGTTGATGGTGCTAGAAGAATTTGAGAAGAAATACTCCTGTATTGAGTTGTATCGTGTGGTGTATTTTTTAAAAAATTTGATTTAGCATTCATATTTTCCATCTTATTCCCAATTAAAAGTATGCAGATTATTTGCCCAAATCTTCTTCAGATTCAGCATTTGTTCTTTGCCAGTCTCATTTTCATCTTCTTCCATGGTTCCACAGAAGCTTTGTTTCTTGGGCAAGCAGAAAAATTAAATTGTACCTATTTTGTATATGTGAGATGTTTAAATAAATTGTGAAAAAAATGAAATAAAGCATGTTTGGTTTTCCAAAAGAA |
| PSIV | Plautia stali intestine virus RNA for nonstructural polyprotein, capsid protein precursor, complete cds NC_003779.1 | AATGCCTTGCATTGGCAGTAGAGTTTTTCCCCAGGGAGCTTCACTGTCTGGGTTTTCTCTACTTATCACCTCCTCACTTGGACGGAGGCTCTTCCGGTTAACAGCTTCACTGTTGATCTGACAGAATGTTGGTACATAGCTTCACTATGTATCAAAGTTTCATTTTTGC |
| SFV | Semliki forest virus NC_003215.1 | GTTAGGGTAGGCAATGGCATTGATATAGCAAGAAAATTGAAAACAGAAAAAGTTAGGGTAAGCAATGGCATATAACCATAACTGTATAACTTGTAACAAAGCGCAACAAGACCTGCGCAATTGGCCCCGTGGTCCGCCTCACGGAAACTCGGGGCAACTCATATTGACACATTAATTGGCAATAATTGGAAGCTTACATAAGCTTAATTCGACGAATAATTGGATTTTTATTTTATTTTGCAATTGGTTTTTAATATTTCC |

| | | |
|---|---|---|
| ZIKV | Zika virus, complete genome NC_012532.1 | GCACCAATTTTAGTGTTGTCAGGCCTGCTAGTCAGCCACAGTTTGGGGAAAGCTGTGCAGCCTGTAACCCCCCCAGGAGAAGCTGGGAAACCAAGCTCATAGTCAGGCCGAGAACGCCATGGCACGGAAGAAGCCATGCTGCCTGTGAGCCCCTCAGAGGACACTGAGTCAAAAAACCCCACGCGCTTGGAAGCGCAGGATGGGAAAAGAAGGTGGCGACCTTCCCCACCCTTCAATCTGGGGCCTGAACTGGAGACTAGCTGTGAATCTCCAGCAGAGGGACTAGTGGTTAGAGGAGACCCCCCGGAAAACGCAAAACAGCATATTGACGTGGGAAAGACCAGAGACTCCATGAGTTTCCACCACGCTGGCCGCCAGGCACAGATCGCCGAACTTCGGCGGCCGGTGTGGGGAAATCCATGGTTTCT |
| EMCV | Encephalomyocarditis virus strain pEC9 DQ288856.1 | TAGTGTAGTCACTGGCACAACGCGTTACCCGGTAAGCCAATCGGGTATACACGGTCGTCATACTGCAGACAGGGTTCTTCTACTTTGCAAGATAGTCTAGAGTAGTAAAATAAATAGATAGAG |
| Modrena | Synthetic construct clone WITO4160.27.ENV.gp145.G153E envelope glycoprotein gene, complete cds MZ362873.1 | GCTGGAGCCTCGGTGGCCTAGCTTCTTGCCCCTTGGGCCTCCCCCCAGCCCCTCCTCCCCTTCCTGCACCCGTACCCCCGTGGTCTTTGAATAAAGTCTGAGTGGGCGGC |
